# Supplementary material for: Dermal injury drives a skin to gut axis that disrupts the intestinal microbiome and intestinal immune homeostasis in mice
Source: Nat Commun. 2024 Apr 8;15:3009. doi: 10.1038/s41467-024-47072-3 (PMC11001995; doi:10.1038/s41467-024-47072-3)
Supplement: Supplementary file 1 — Supplementary Information [file 41467_2024_47072_MOESM1_ESM.pdf]

Supplemental Figures

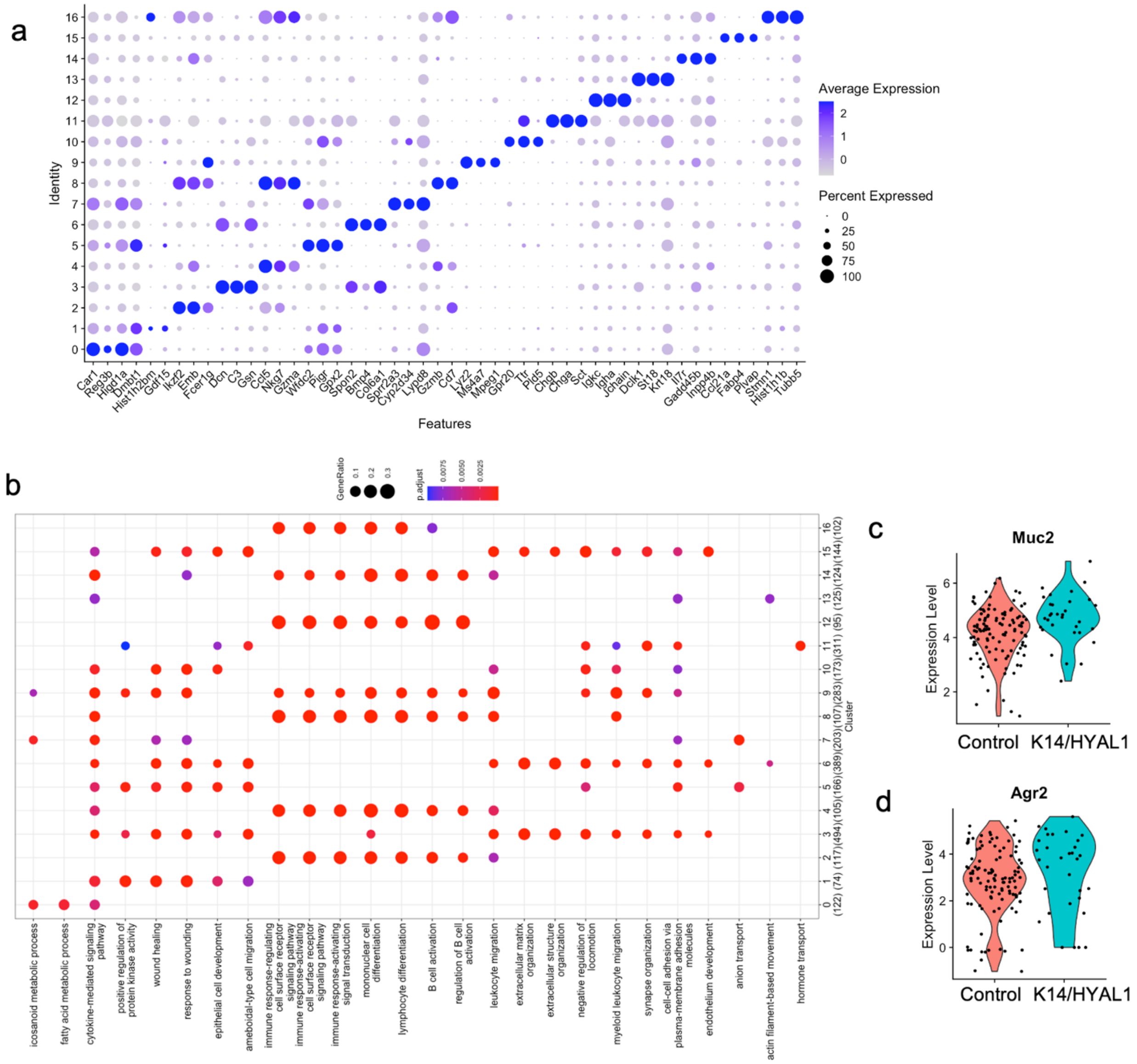

Supplementary Figure 1

Single cell RNA Sequencing of colon in *K14/HYAL1* mice

Changes in gene expression in the colon of *K14/HYAL1* mice are detected by scRNASeq. a. Top 3 differentially expressing genes in each cluster. b. GO terms from each cluster. c. Violin plot of *Muc2*, and d- *Agr2* from cluster 10.

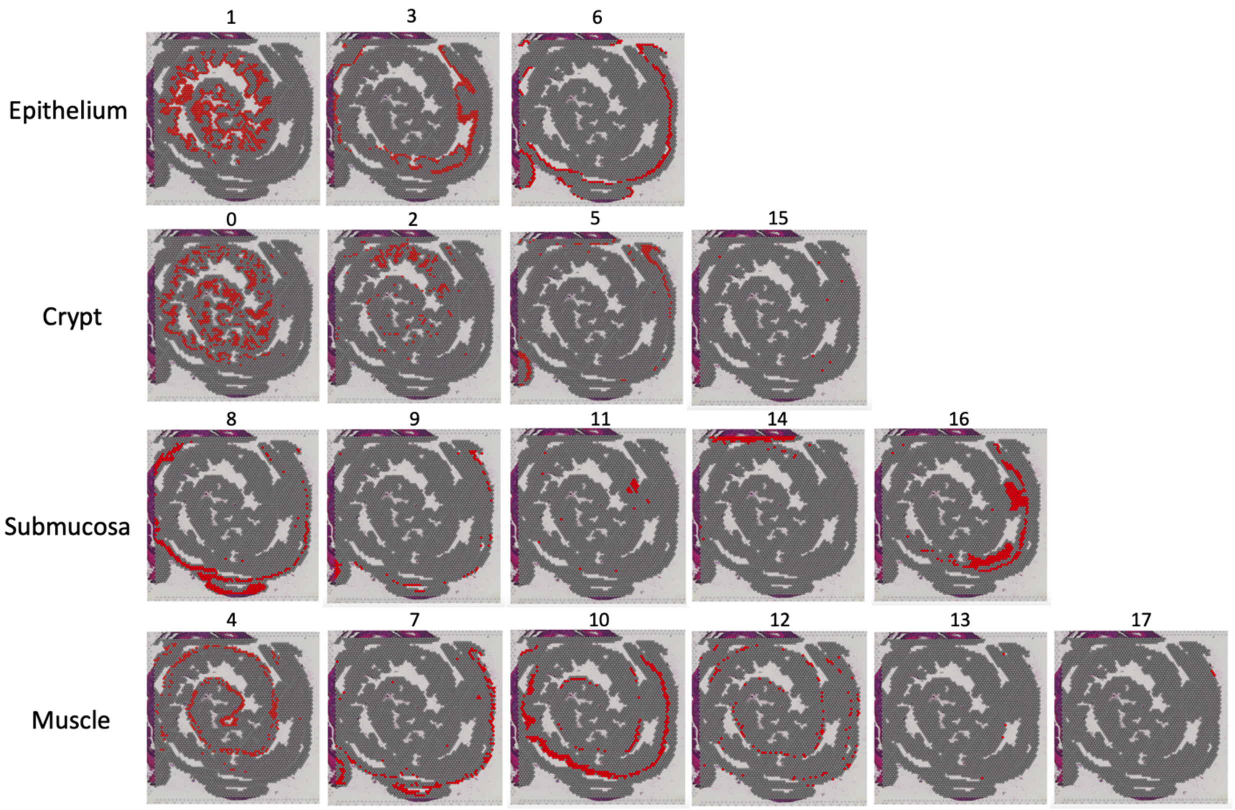

Supplementary Figure 2

Location of cell clusters in the intestine as identified by spatial sequencing.
Spatial plot of identified numbered clusters in Red in each tissue as defined by UMAP plot shown in Figure 1F in *K14/HYAL1*.

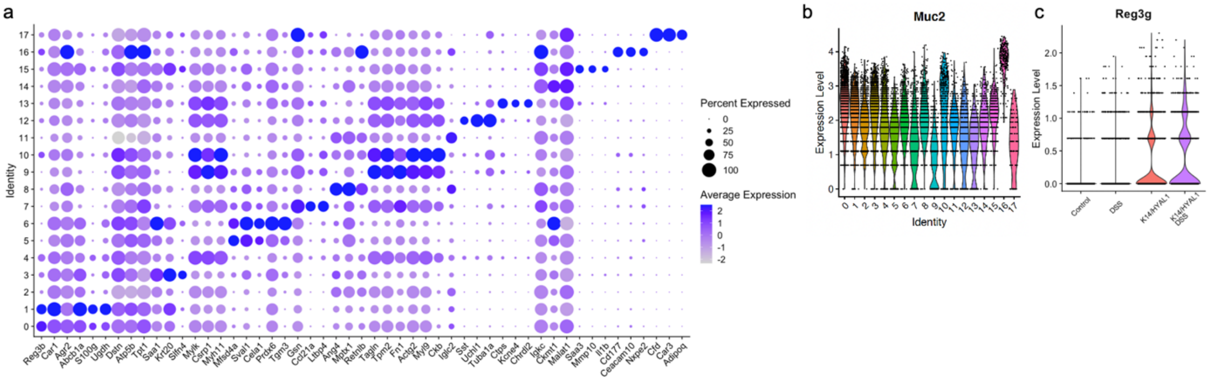

Supplementary Figure 3

Spatial RNA Sequencing of mouse colon after expression of *HYAL1* in the skin and challenge with DSS

a. Top 3 differentially expressing genes in each cluster. b. Violin plot of *Muc2* in each cluster. c. Violin plot of *Reg3g* in each group.

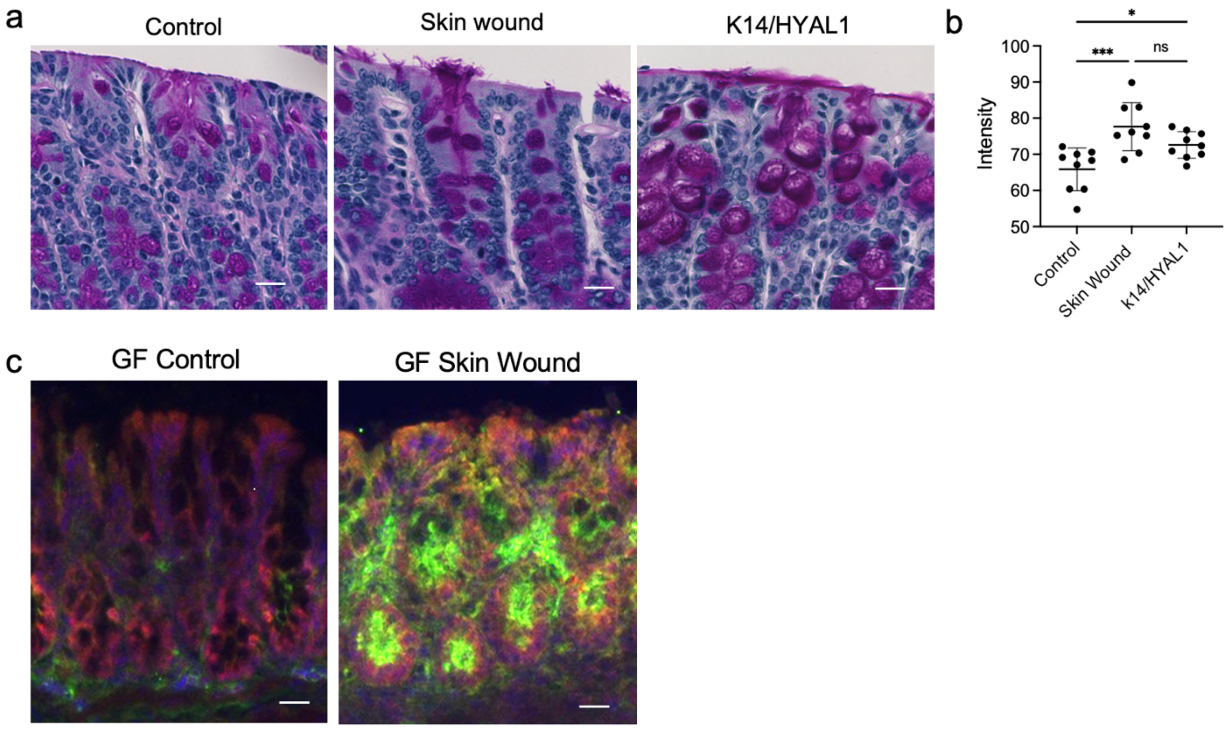

Supplementary Figure 4

Mucin and Reg3g production in the skin after HA digestion

a. Representative High magnification image of periodic acid–Schiff (PAS) staining in the transverse colon. (Scale bar: 25 microns) b. Intensity of PAS staining from Control, Skin wound, *K14/HYAL1*. (n=9 independent biological replicates per group) c. High magnification of immunofluorescent staining of the colon. (Muc2: Green, Reg3g: Red DAPI: Blue. Scale bar: 25 microns.) Statistical significance was determined using ordinary one-way ANOVA and Tukey's multiple comparison two-sided test. Error bars indicate mean ± SD; * $P<0.05$, ** $P < 0.01$, *** $P<0.001$. Each experiment was repeated at least 3 times. Source data are provided as a Source Data file.

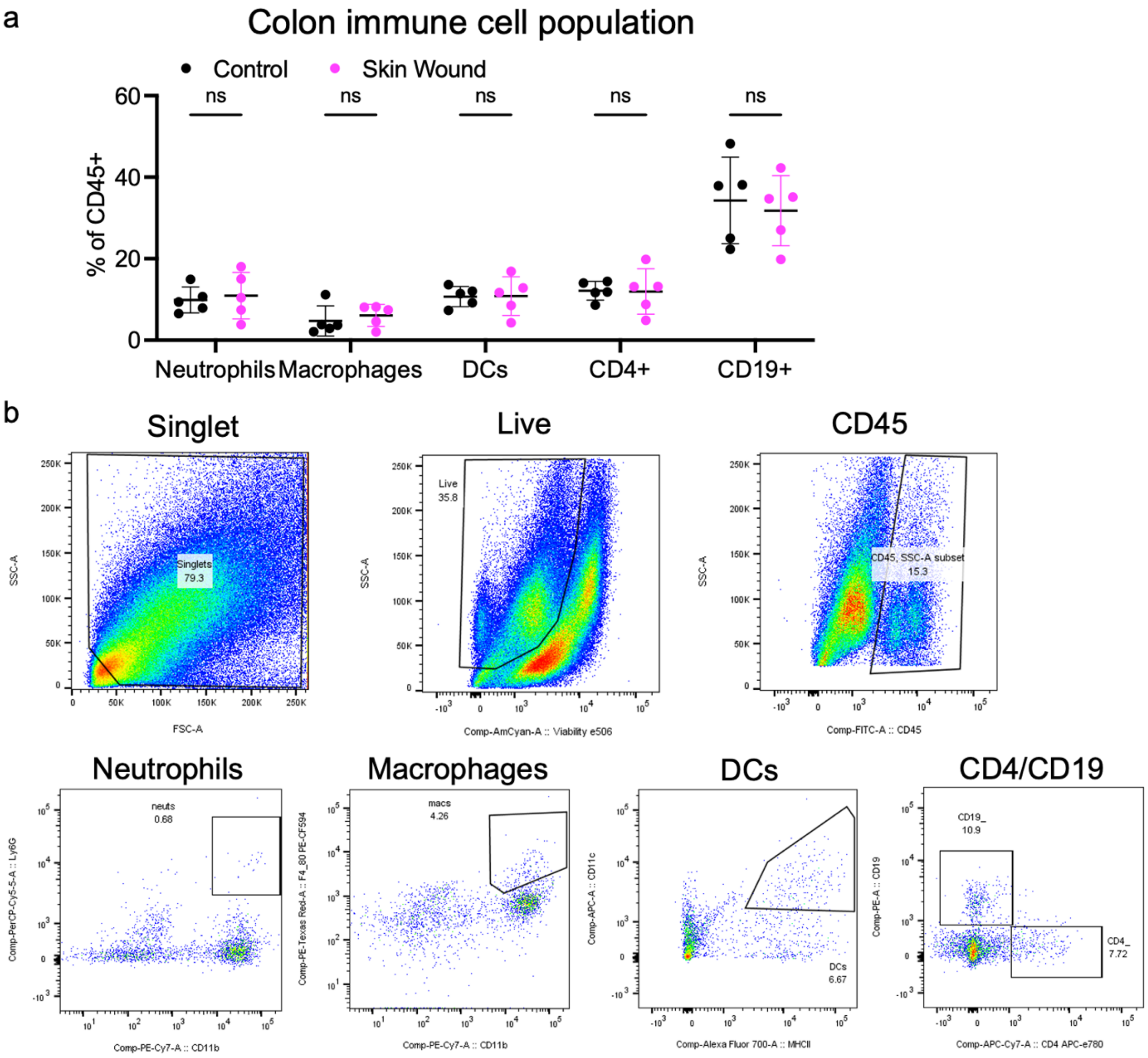

Supplementary Figure 5

FACS analysis of immunocytes in the colon of mice following skin wounding

a. % of neutrophils, macrophages, dendritic cells (DCs), CD4+ and CD19+ cell from total CD45+ cells measured by FACS from the colon of control and littermates 48 hours after skin injury (n=5 independent biological replicates per group). b. FACS gating strategy. Live singlets with CD45+ cells are gated as immune cells. Statistical significance was determined using ordinary one-way ANOVA and Tukey's multiple comparison test. Error bars indicate mean ± SD; * P<0.05, ** P < 0.01, *** P<0.001. Each experiment was repeated at least 3 times. Source data are provided as a Source Data file.

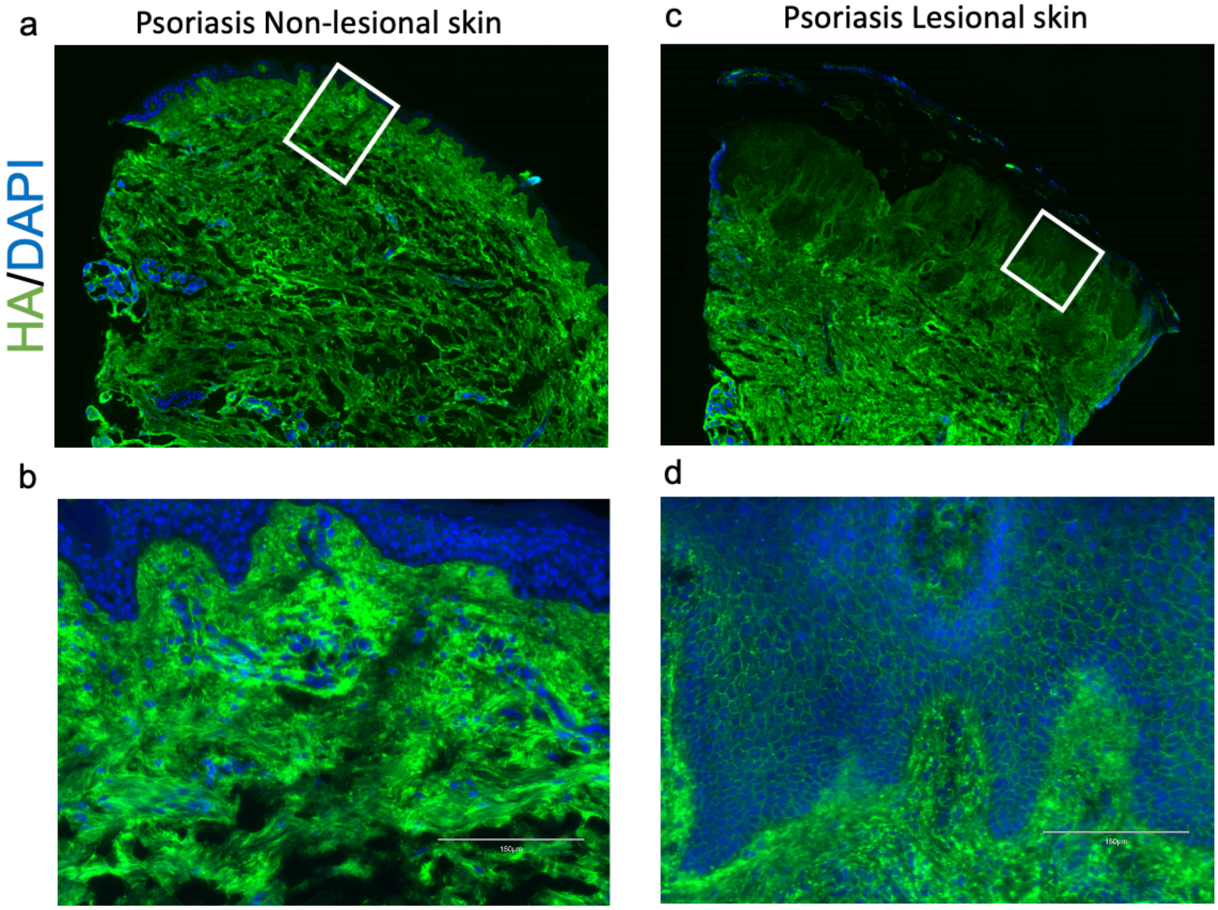

Supplementary Figure 6

Hyaluronan is decreased in Psoriatic lesional skin.

Staining of Hyaluronan using fluorescent hyaluronan binding protein demonstrates loss of hyaluronan in the reticular dermis associated with local inflammation present in psoriasis lesional skin. a. Low power magnification (10X) of non-lesional skin. b. High power magnification (40X) of the dermis of non-lesional skin at area indicated by box in a. c. Low power magnification (10X) of psoriasis lesional skin. d. High power magnification (40X) of the dermis of lesional skin at area indicated by box in c. (HA: Green, DAPI: Blue). Scale bar: 150 microns.

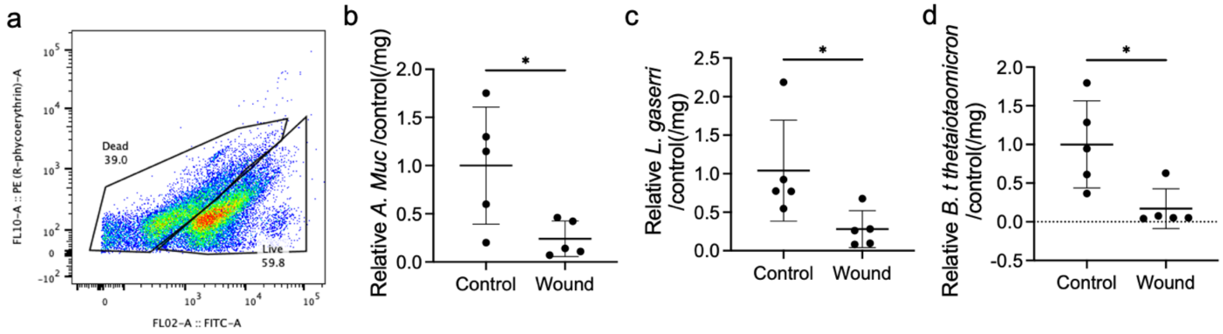

Supplementary Figure 7

Analysis of bacteria from feces following skin wounding

a. FACS gating strategy. b to d. qPCR measurement of relative abundance of 16S rDNA per mg feces of *A. muciniphila* (a), *L. gasseri* (b), and *B. thetaiotaomicron*(c) (n=5 independent biological replicates per group). Statistical significance was determined using Student's unpaired two-tailed t test. Error bars indicate mean ± SD; * P<0.05, ** P < 0.01, *** P<0.001. Each experiment was repeated at least 3 times. Source data are provided as a Source Data file.

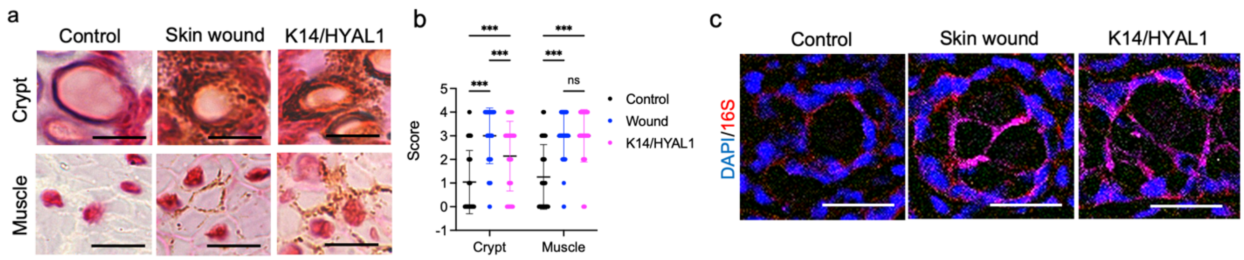

Supplementary Figure 8

Gram staining of colon following in *K14/HYAL1* mice or in mice after skin wounds.

a. Gram staining of bacteria in the transverse colon at high magnification of crypt and muscle layer under the crypt structure. (Scale bar: 25 microns.). b. Histological quantification of the amount of gram staining. (n=5 independent biological replicates per group) c. in situ hybridization assay of bacterial 16S rDNA in the colon at high magnification of crypt structure. (Scale bar: 25 microns. 16S: Red, DAPI: Blue) Statistical significance was determined using ordinary one-way ANOVA and Tukey's multiple comparison two-sided test. Error bars indicate mean ± SD; * $P<0.05$, ** $P < 0.01$, *** $P<0.001$. Each experiment was repeated at least 3 times. Source data are provided as a Source Data file.

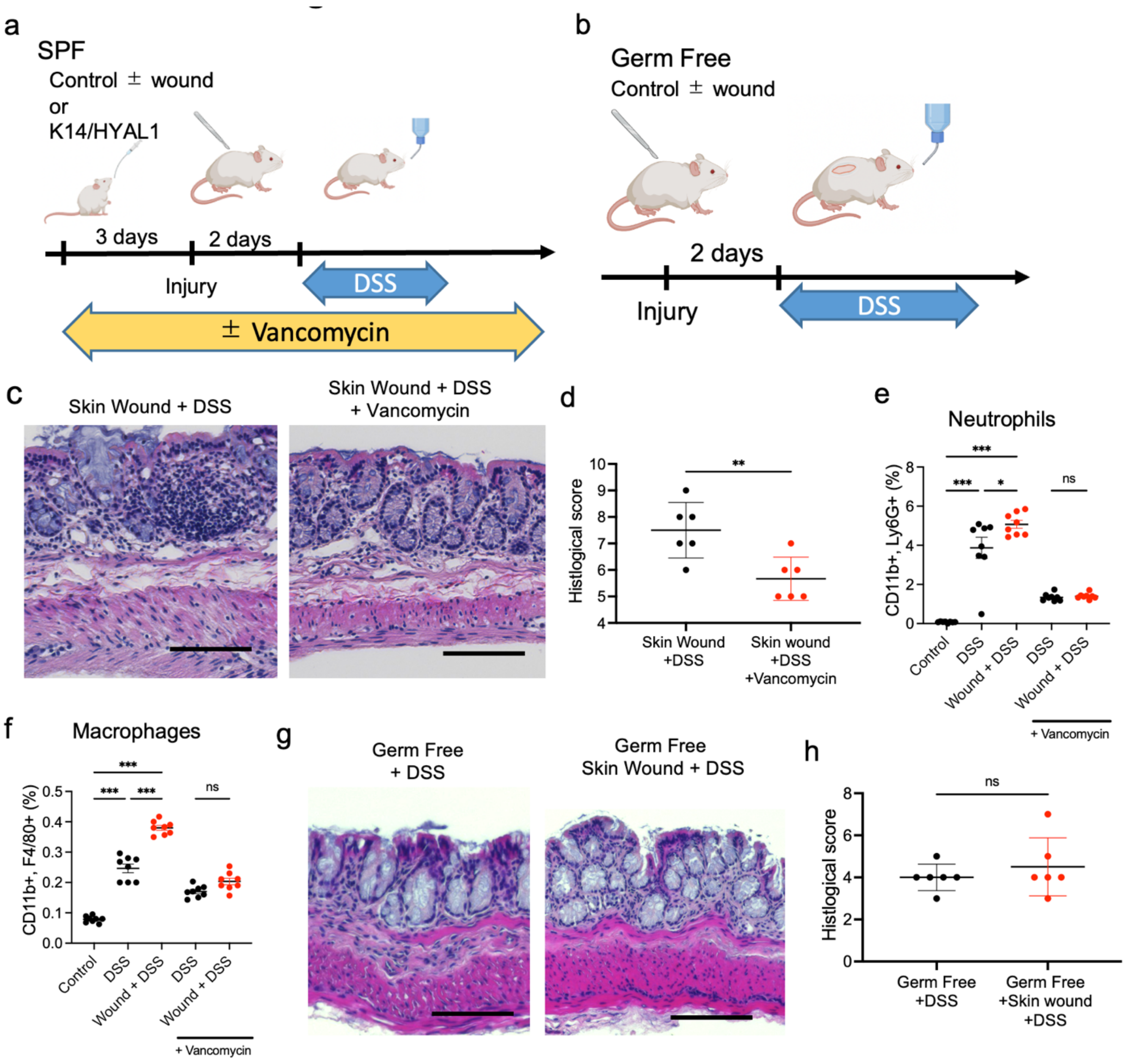

Supplementary Figure 9

The intestinal microbiome is required for the skin to increase susceptibility to DSS colitis
a and b, Schematics of the experimental design created with BioRender.com. a- Mice housed under SPF conditions were administered oral vancomycin (50mg/kg) for 5 days prior, and skin wounding performed 2 days prior, to challenge with DSS in water b- Skin wounding was performed in gnotobiotic (Germ Free) mice 2 days prior to challenge with DSS. C and d. Histological images of the distal colon SPF mice treated with Vancomycin and Histological score (n=6 independent biological replicates per group). e and f. % neutrophils and macrophages measured by FACS from the colon of SPF mice (n=8 independent biological replicates per group). g and h. Histological images of the distal colon from germ-free mice. and Histological score (n=6 independent biological replicates per group). Scale bar: 50 microns. Statistical significance was determined using ordinary one-way ANOVA and Tukey's multiple comparison two-sided test. Error bars indicate mean ± SD; * P<0.05, ** P < 0.01, *** P<0.001. Each experiment was repeated at least 3 times. Source data are provided as a Source Data file.

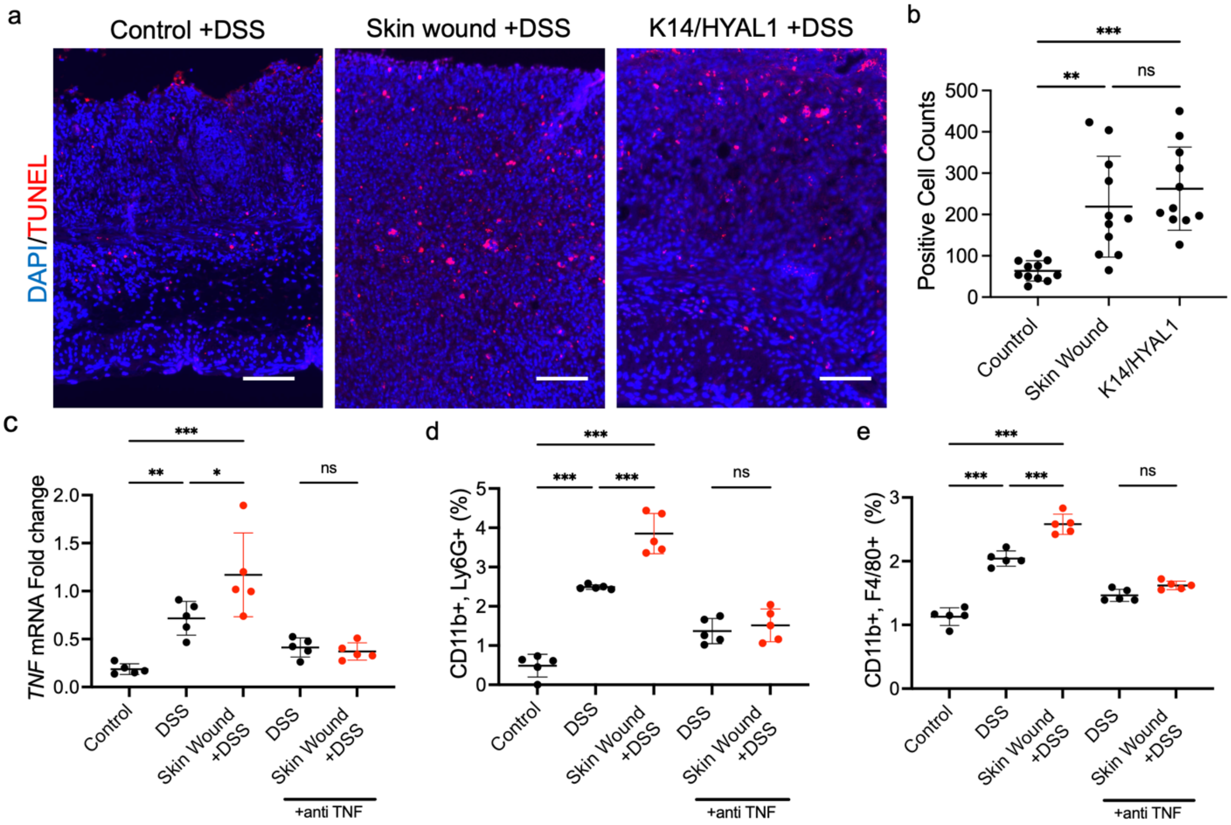

Supplementary Figure 10.

The capacity of the skin to promote DSS colitis is dependent on TNF in the gut

a. Tunel staining of the colon of mice after DSS challenge to control, skin wound or *K14/HYAL1* mice. (Scale bar: 25 microns. TUNEL: Red, DAPI: Blue) b. The number of apoptosis cells in the image (n=11 independent biological replicates per group). c. *TNF* mRNA expression in the colon of mice after DSS challenge following skin wounding with or without anti-TNF treatment by XT3.11 (n=5 independent biological replicates per group). d and e. % neutrophils and macrophages measured by FACS (n=5 independent biological replicates per group). Scale bar: 50 microns. Statistical significance was determined using ordinary one-way ANOVA and Tukey's multiple comparison two-sided test. Error bars indicate mean ± SD; * P<0.05, ** P < 0.01, *** P<0.001. Each experiment was repeated at least 3 times. Source data are provided as a Source Data file.

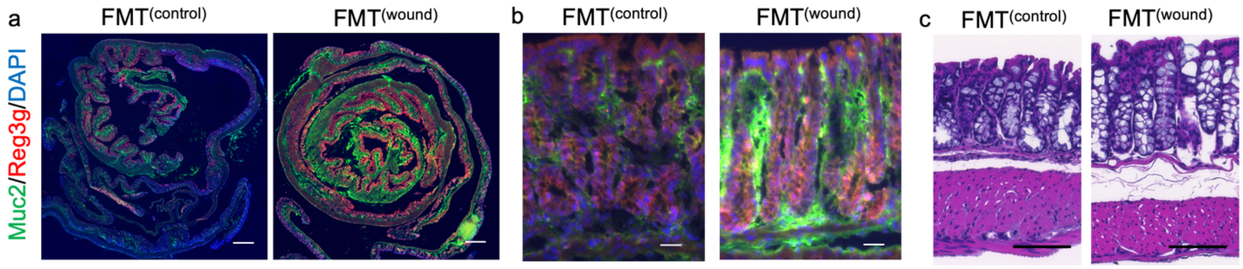

Supplementary Figure 11

Transfer of fecal microbes from mice with skin wounds induces Muc2 and Reg3g in the intestine.

a. Immunofluorescent staining of the Whole colon. (Muc2: Green, Reg3g: Red DAPI: Blue. Scale bar: 1000 microns.). b. High magnification of crypt structure. (Scale bar: 25 microns.) c. Histological images of the distal colon following fecal microbiome transfer from mice with skin wounds and control mice.

Supplementary Table 1: List of primer sequences used in this study

	**Primer name**	**Sequence (5' – 3')**
**Mouse**	Reg3g forward	CGTGCCTATGGCTCCTATTGCT
	Reg3g reverse	TTCAGCGCCACTGAGCACAGAC
	TNF forward	ACTTCGGGGTGATCGGTCCCC
	TNF reverse	TGGTTTGCTACGACGTGGGCTAC
	IL6 forward	ACAAAGCCAGAGTCCTTCAGAGAGA
	IL6 reverse	AGCCACTCCTTCTGTGACTCCAG
**Human**	Reg3A forward	TAATGTGAGGTTACCCTATG
	Reg3A reverse	GAGGAAGAAACAGAAGAAAG
**Bacterial**	Univ16S-1048-F	GTGSTGCAYGGYTGTCGTCA
	Univ16S-1175-R	ACGTCRTCCMCACCTTCCTC
	A. muciniphila Forward	CAGCACGTGAAGGTGGGGAC
	A. muciniphila Reverse	CCTTGCGGTTGGCTTCAGAT
	L. gasseri Forward	AAGGGCGCACGGTGAATGCCT
	L. gasseri Reverse	TGCTATCGCTTCAAGTGCTT
	B. thetaiotaomicron Forward	GCAAACTGGAGATGGCGA
	B. thetaiotaomicron Reverse	AAGGTTTGGTGAGCCGTTA
