# Supplementary material for: Dermal injury drives a skin to gut axis that disrupts the intestinal microbiome and intestinal immune homeostasis in mice
Source: Nat Commun. 2024 Apr 8;15:3009. doi: 10.1038/s41467-024-47072-3 (PMC11001995; doi:10.1038/s41467-024-47072-3)
Supplement: Supplementary file 3 — Reporting Summary [file 41467_2024_47072_MOESM3_ESM.pdf]

nature portfolio

Corresponding author(s): Tatsuya Dokoshi, Yang Chen, Kellen J. Cavagnero, Gibraan Rahman, Daniel Hakim, Samantha Brinton, Hana Schwarz, Elizabeth A. Brown, Alan O'Neill, Yoshiyuki Nakamura, Fengwu Li, Nita H. Salzman, Rob Knight, Richard L. Gallo

Last updated by author(s): Feb 22, 2024

# Reporting Summary

Nature Portfolio wishes to improve the reproducibility of the work that we publish. This form provides structure for consistency and transparency in reporting. For further information on Nature Portfolio policies, see our Editorial Policies and the Editorial Policy Checklist.

## Statistics

For all statistical analyses, confirm that the following items are present in the figure legend, table legend, main text, or Methods section.

| n/a | Confirmed | |
|---|---|---|
| ☐ | ☒ | The exact sample size (*n*) for each experimental group/condition, given as a discrete number and unit of measurement |
| ☐ | ☒ | A statement on whether measurements were taken from distinct samples or whether the same sample was measured repeatedly |
| ☐ | ☒ | The statistical test(s) used AND whether they are one- or two-sided<br>*Only common tests should be described solely by name; describe more complex techniques in the Methods section.* |
| ☐ | ☒ | A description of all covariates tested |
| ☐ | ☒ | A description of any assumptions or corrections, such as tests of normality and adjustment for multiple comparisons |
| ☐ | ☒ | A full description of the statistical parameters including central tendency (e.g. means) or other basic estimates (e.g. regression coefficient) AND variation (e.g. standard deviation) or associated estimates of uncertainty (e.g. confidence intervals) |
| ☐ | ☒ | For null hypothesis testing, the test statistic (e.g. *F*, *t*, *r*) with confidence intervals, effect sizes, degrees of freedom and *P* value noted<br>*Give P values as exact values whenever suitable.* |
| ☒ | ☐ | For Bayesian analysis, information on the choice of priors and Markov chain Monte Carlo settings |
| ☐ | ☒ | For hierarchical and complex designs, identification of the appropriate level for tests and full reporting of outcomes |
| ☒ | ☐ | Estimates of effect sizes (e.g. Cohen's *d*, Pearson's *r*), indicating how they were calculated |

*Our web collection on statistics for biologists contains articles on many of the points above.*

## Software and code

Policy information about availability of computer code

| | |
|---|---|
| Data collection | no software was used for data collection in this study |
| Data analysis | Prism 10, R studio(2023.12.1+402), All the code related to the data analysis is deposited in the following link. https://github.com/yangchen2/gallo_skin-gut_axis |

For manuscripts utilizing custom algorithms or software that are central to the research but not yet described in published literature, software must be made available to editors and reviewers. We strongly encourage code deposition in a community repository (e.g. GitHub). See the Nature Portfolio guidelines for submitting code & software for further information.

## Data

Policy information about availability of data

All manuscripts must include a data availability statement. This statement should provide the following information, where applicable:

- Accession codes, unique identifiers, or web links for publicly available datasets
- A description of any restrictions on data availability
- For clinical datasets or third party data, please ensure that the statement adheres to our policy

The Single cell and spatial sequencing data generated in this study have been deposited in the GEO database under accession code (GSE227836) [https://

www.ncbi.nlm.nih.gov/geo/query/acc.cgi?acc=GSM7109548]. The microbiome data generated in this study is available on QIITA, study ID 14365. [https://qiita.ucsd.edu/study/description/14365], BioProject ID: PRJNA1003965.[https://www.ebi.ac.uk/ena/browser/view/PRJNA1003965]
The data generated in this study are provided in the Supplementary Information/Source Data file.

## Research involving human participants, their data, or biological material

Policy information about studies with human participants or human data. See also policy information about sex, gender (identity/presentation), and sexual orientation and race, ethnicity and racism.

| | |
|---|---|
| Reporting on sex and gender | This information is not collected in this study. |
| Reporting on race, ethnicity, or other socially relevant groupings | This information is not collected in this study. |
| Population characteristics | Participants are currently diagnosed with psoriasis, exhibiting active inflammatory lesion. |
| Recruitment | Participants are currently diagnosed with psoriasis, exhibiting mild symptoms. |
| Ethics oversight | Ethical oversight for this study was conducted by the Institutional Review Board (IRB) at the University of California San Diego (UCSD). All procedures involving human participants were reviewed and approved by the UCSD IRB to ensure compliance with ethical standards and protection of participant rights. Written informed consent was obtained from all participants prior to their inclusion in the study. |

Note that full information on the approval of the study protocol must also be provided in the manuscript.

# Field-specific reporting

Please select the one below that is the best fit for your research. If you are not sure, read the appropriate sections before making your selection.

[x] Life sciences [ ] Behavioural & social sciences [ ] Ecological, evolutionary & environmental sciences

For a reference copy of the document with all sections, see nature.com/documents/nr-reporting-summary-flat.pdf

# Life sciences study design

All studies must disclose on these points even when the disclosure is negative.

| | |
|---|---|
| Sample size | For spatial and single-cell sequencing, we randomly selected mice from each group and ran one set of samples.<br>We conducted a middle-sized cohort study to analyze the fecal microbiome of mice, using complete sets of co-housed litter-mates as controls. This study included a similar number of samples compared to previous studies in the field such as the study by [Ferdinando Scavizzi et al., 2021] published in anim microbiome.<br>Sample sizes for mouse experiments were determined by the current standard used for mice, based on the minimal amount of mice required to detect significance with an alpha rate set at .05 in a standardly powered experiment. |
| Data exclusions | Mice that died or experienced severe complications as a result of the procedural techniques were excluded from the analysis. |
| Replication | We conducted each experimental model at least three times, using the number of mice shown, and representative data was shown in this article. |
| Randomization | In this study, animals were assigned randomly to experimental and control groups using a random number generator. |
| Blinding | The investigators were blinded during data analysis. |

# Reporting for specific materials, systems and methods

We require information from authors about some types of materials, experimental systems and methods used in many studies. Here, indicate whether each material, system or method listed is relevant to your study. If you are not sure if a list item applies to your research, read the appropriate section before selecting a response.

## Materials & experimental systems

| n/a | Involved in the study | |
|---|---|---|
| ☐ | ☒ | Antibodies |
| ☐ | ☒ | Eukaryotic cell lines |
| ☒ | ☐ | Palaeontology and archaeology |
| ☐ | ☒ | Animals and other organisms |
| ☒ | ☐ | Clinical data |
| ☒ | ☐ | Dual use research of concern |
| ☒ | ☐ | Plants |

## Methods

| n/a | Involved in the study | |
|---|---|---|
| ☒ | ☐ | ChIP-seq |
| ☐ | ☒ | Flow cytometry |
| ☒ | ☐ | MRI-based neuroimaging |

# Antibodies

Antibodies used: HABP(#385911, EMD Millipore), Muc2(#PIMA512345, Fisher), Reg3g (#PA5-50450, Thermo Fisher)

Validation: References are available. HABP(#385911) :https://doi.org/10.3390/cancers12051325. Muc2(#PIMA512345): https://doi.org/10.1128/AEM.01061-17. Reg3g (#PA5-50450):https://doi.org/10.1038/s41467-020-19691-z

# Eukaryotic cell lines

Policy information about cell lines and Sex and Gender in Research

Cell line source(s): HT-29 human CRC cells were purchased from American Type Culture Collection.

Authentication: None of the cell line used in this study is not authenticated.

Mycoplasma contamination: the cell lines were not tested for mycoplasma contamination.

Commonly misidentified lines (See ICLAC register): We don't use any commonly misidentified lines.

# Animals and other research organisms

Policy information about studies involving animals; ARRIVE guidelines recommended for reporting animal research, and Sex and Gender in Research

Laboratory animals: Wildtype mice (C57BL/6 mice) and K14-cre transgenic mice were obtained from The Jackson Laboratory. K14-cre transgenic mice were bred with Hyal1 mice for the generation of K14-cre Hyal1 mice. 8 to 12 weeks age mice are used for experiments.

Wild animals: no wild animals were used in the study.

Reporting on sex: We use 1:1 ratio of male and female mice n this study.

Field-collected samples: no field collected samples were used in the study.

Ethics oversight: All animal experiments were approved by the University of California, San Diego, Institutional Animal Care(IACUC).

Note that full information on the approval of the study protocol must also be provided in the manuscript.

# Plants

Seed stocks: N/A

Novel plant genotypes: N/A

Authentication: N/A

# Flow Cytometry

## Plots

Confirm that:

- [x] The axis labels state the marker and fluorochrome used (e.g. CD4-FITC).
- [x] The axis scales are clearly visible. Include numbers along axes only for bottom left plot of group (a 'group' is an analysis of identical markers).
- [x] All plots are contour plots with outliers or pseudocolor plots.
- [x] A numerical value for number of cells or percentage (with statistics) is provided.

## Methodology

| | |
|---|---|
| Sample preparation | Colon collected from control or DSS-treated mice was cut into small pieces then digested with 2.5 mg/mL Collagenase D and 30 ng/mL DNAse1 for 40 minites at 37°C then filtered through a 70 µm filter to generate single cell suspension for FACS analyses. |
| Instrument | BD FACSCanto RUO - Orange |
| Software | FlowJo V10 |
| Cell population abundance | Cell population number is included in the article. |
| Gating strategy | The article presents the gating strategy used after FSC/SSC gating for singlets and live cells. |

- [x] Tick this box to confirm that a figure exemplifying the gating strategy is provided in the Supplementary Information.
